# A Retrospective Analysis on Risk Factors for 30-day Readmission Rates in Patients Living With HIV and Severe Major Depression Disorder

**DOI:** 10.7759/cureus.15894

**Published:** 2021-06-24

**Authors:** Sindhura Kompella, Joseph Ikekwere, Clara Alvarez, Ian H Rutkofsky

**Affiliations:** 1 Psychiatry, Aventura Hospital and Medical Center, Aventura, USA; 2 Psychiatry/Addiction, University of Illinois at Chicago, Chicago, USA

**Keywords:** hiv, minority, hospital readmission rate, major depressive disorder, people living with hiv/aids

## Abstract

Background

Major depression disorder (MDD) is the most common psychiatric comorbidity in patients living with HIV (PLWHIV). The prevalence rate of MDD is higher in PLWHIV in comparison to the general population. In our study, we focus specifically on the 30-day readmission rate of PLWHIV and severe major depression.

Methods

The Health Care Agency (HCA) databank was used to conduct a retrospective study on PLWHIV and severe MDD. Keywords such as HIV, severe MDD, CD4, viral load were used to identify the data. 30-day readmission rate is studied in PLWHIV and severe MDD (N=143). Variables such as age, sex, gender, adherence to antiretroviral medications, cluster of differentiation 4 (CD4), and viral load were studied in this population. *Diagnostic and Statistical Manual of Mental Disorders, 5th Edition *(DSM-5) criteria were used to diagnose severe MDD in PLWHIV. An antiretroviral therapy (ART) medication list was used to analyze adherence in this population group. Geographical locations were identified using urbanization codes.

Results

Logistic regression analysis for the 30-day readmission rate in PLWHIV was found to be higher in the older age group (p<0.01). Caucasian population (p<0.01) and rural areas (p<0.01), ART non-adherence (p<0.05), and severe major depression were also found to be significant in this population (p<0.01).

Conclusion

As more patients live longer with HIV/AIDS, it gives rise to illnesses such as anxiety, depression, and cognitive impairment. Thus, it is important to identify severe depression in PLWHIV since it can have an impact on rates of hospitalization, morbidity/mortality, and the financial burden, specifically within 30-days of discharge.

## Introduction

The 30-day readmission rates have become an integral quality care metric across healthcare since they may be associated with an increase in financial costs, psychosocial stressors, and mortality [1]. People living with HIV (PLWHIV) have been shown to have higher rates of readmission compared to the general population, despite the advances in HIV treatment in the form of highly active antiretroviral therapy (HAART) [2].

The earlier years of the Acquired Immunodeficiency Syndrome (AIDS) epidemic resulted in hospitalizations and readmissions based on the novelty of the disease pathology and lack of efficacious treatment modalities. While the HAART era in the 1990s ushered in a period of steady decline associated with AIDS-related complications, it also resulted in an increase in the rates of hospitalizations and readmissions due to an increase in mental healthcare burden in this population group [3].

While studies have been conducted on readmission rates in PLWHIV focusing on preventative measures and management techniques, they have been found too broad in their scope of review [3]. Moreover, these studies are lacking in delineating which specific risk factors are associated with mental illnesses in HIV or 30-day readmission rates in this population group.

Since depression is the most prevalent mental health disorder in PLWHIV, this study aims to study severe major depression disorder (MDD) in PLWHIV [3], while outlining the contributing risk factors for 30-day readmission rates in this population group with co-occurring severe MDD [4].

## Materials and methods

This study was conducted in accordance with the Declaration of Helsinki and appropriate Institutional Review Board (IRB) approval was obtained. A waiver of informed consent was deemed appropriate by the IRB. We utilized the Health Care Agency (HCA) enterprise-level database to retrospectively study (2015-2020) the impact of depression on PLWHIV. Inclusion criteria included demographics such as gender: male or female; age: 18-85 years, and ethnicity: Caucasian, African American, Hispanic, and Other. Only inpatient hospital discharges and readmission data is obtained using the keywords HIV, MDD, CD4 count, and viral load.

Each patient’s viral load was evaluated as a binary outcome with detectable viral load copies ranging from >20 copies/ml to undetectable. CD4 count <500 was used as an inclusionary criterion for determining HIV. CD4 count <200 was determined as AIDS. The antiretroviral therapy (ART) medications analyzed comprised any available single-dose, combination medication, or ART cocktail comprising of three or more medications including integrase strand transfer inhibitors (INSTIs), nucleoside/nucleotide reverse transcriptase inhibitors (NRTIs), non-nucleoside reverse transcriptase inhibitors (NNRTIs), cytochrome P4503A (CYP3A) inhibitors, protease Inhibitors (PIs), fusion inhibitors (FIs), post-attachment inhibitors, chemokine co-receptor antagonists (CCR5 antagonists), and entry inhibitors.

*Diagnostic and Statistical Manual of Mental Disorders, 5th Edition *(DSM-5) criteria were used: F33.2 [5], while ICD 10 codes: F33.0, F33.1, F33.3, F33.4, F33.9 (mild, moderate severity, psychotic features, etc) were excluded. The index hospitalizations determined were day 1 and day 30 and included hospitalizations for patients being treated for HIV/AIDS. Urbanization codes were established utilizing the Environmental Systems Research Institute (ESRI) demographics tapestry segmentation system and these codes were used to distinguish urban and rural geographical localities into further sub-geographic locations consisting of metro cities, principal urban centers, rural, semirural, suburban periphery, and urban periphery [6]. Insurance status was also reviewed as private, government, or no insurance. The insurance variable was used as a measure of socioeconomic status. The data was analyzed using binary logistic regression analysis to predict 30-day readmission rates in PLWHIV and severe MDD.

## Results

In our study, there were a total of n = 143 PLWHIV and severe MDD identified who were readmitted within 30 days of discharge. The mean age for this population group was found to be 60 years (Table 1). Increasing age is found to be a significant risk factor for readmission within 30 days and the Caucasian population is more likely to be readmitted within 30 days compared to other ethnicities (p<0.001).

**Table 1:** Logistic regression for 30-day readmission rate in PLWHIV, severe MDD: demographics: age, gender, and race; ART adherence. ART, antiretroviral therapy; EXP, exponential; sig, significant; PLHIV, people living with HIV; MDD, major depression disorder

Demographics	Sig value (p)	95% CI for EXP Lower	95% CI for EXP Upper
Age	0.002	0.987	0.997
Gender	0.730	0.708	6.079
Race (Caucasian)	0.001	0.473	0.616
ART non-adherence	0.005	0.190	0.593

Controlling for the other variables in the logistic regression analysis, we also found that patients with ART non-adherence were more likely to be readmitted within 30 days of discharge when compared to patients with adherence to ART (p<0.05).

Additionally, uninsured patients and government insurance were more likely to be readmitted in a 30-day interval compared to those with private insurance (p<0.0001) (Table 2). Also, patients living in suburban periphery areas (urbanization codes: R, SP) were also more likely to be readmitted within 30 days of discharge in comparison to urban areas (urbanization codes: MC, PUC, SR; p<0.005) (Table 3).

**Table 2:** Logistic regression for 30-day readmission rate in PLWHIV, severe MDD, and insurance status. EXP, exponential; sig, significant; PLHIV, people living with HIV; MDD, major depression disorder

Insurance status	Sig value (p)	95% CI for EXP Lower	95% CI for EXP Upper
Insurance (Government)	0.000	1.315	1.661
No insurance	0.000	1.315	1.861
Insurance (Private)	0.817	0.780	1.217

**Table 3:** Logistic regression for 30-day readmission rate in PLWHIV, severe MDD, and urbanization codes. Sig, significant; EXP, exponential; MC, metro cities; PUC, principal urban centers; R, rural; SR, suburban rural; SP, suburban periphery; PLHIV, people living with HIV; MDD, major depression disorder

Urbanization codes	Sig value (p)	95% CI for EXP Lower	95% CI for EXP Upper
Urbanization (MC)	0.644	0.891	1.206
Urbanization (PUC)	0.322	0.883	1.459
Urbanization (R)	0.050	0.768	0.813
Urbanization (SR)	0.239	0.924	1.372
Urbanization (SP)	0.006	0.648	0.929

## Discussion

Pharmacological advancements and biopsychosocial approach towards HIV/AIDs treatment have contributed to longer life expectance. However, longevity in survival has increased AIDS-related medical complications and mental illnesses such as anxiety, cognitive impairment, and the most debilitating of mental health pathologies-depression [7]. Our result shows the statistical significance of the 30-day readmission rate among PLWHIV and severe depression. Additionally, below is a summary of some of the risk factors which may contribute to an increase in the 30-day readmission rate in this population group.

Demographics

Age

An increment in age showed an increase in the likelihood for readmission within 30 days in PLWHIV and severe depression. This may be since this population group presents with severe medical comorbidities as a result of the progression of the illness. Moreover, comorbid severe depression can complicate treatment compliance due to feelings of anhedonia or social withdrawal [7].

Race

Our result demonstrated that Caucasian ethnicity was more likely to be readmitted within 30 days in comparison to Black, Hispanic, or other ethnicities among PLWHIV and severe depression. Contrary to the literature review for minorities versus Caucasians, interestingly, our study found a lower likelihood of readmission among the minority groups [8,9]. This may be due to socio-cultural determinants of health including healthcare access barriers, health-seeking beliefs/behaviors and perhaps, an increase in preventative strategies and drug policy initiatives in some minority groups when compared to Caucasians. Nevertheless, it is important to pay close attention to race/ethnicity as a possible risk factor for readmission among this population group.

ART adherence

The non-adherence to ART, when compared to adherence to ART, resulted in a higher likelihood of a 30-day readmission rate (p<0.01). Partly, this can be explained by the co-occurring burden of depression which can make medication compliance an issue due to an increase in despair, isolation, lack of motivation, and sense of neglect [7, 8]. Therefore, it is important to ensure that ART adherence is maintained in PLWHIV and severe depression.

Insurance status

Our result also showed that uninsured patients and patients with government insurance (which is categorized as low-moderate socioeconomic status) were more likely to be readmitted in comparison to patients with private insurance (which is categorized as high socioeconomic status). The increase in readmission rate further reiterates the dire need for better means of access to health care services in lower socioeconomic status, especially since access to these resources would improve the quality of care for under-served population groups and improve medication compliance in PLWHIV and depression [9]. It can also play a major role in decreasing hospitalizations, financial burden, and mortality in this population group [10].

Geographic location

Similarly, rural groups were more likely to be readmitted within 30 days of discharge in comparison to urban/suburban groups in PLWHIV and severe depression. People living in rural localities report lower life satisfaction related to constricted standards of living.

Partly, this may be due to less robust social support structures, limited or nonexistent access to medical and mental health care, or increased social stigmatization related to HIV status as well as mental health services [9, 10]. These factors can lead to maladaptive coping patterns, poor adherence to medications, and an increase in hospitalizations [11]. Hence, it important to screen for severe depression in this population group to decrease the readmission rate within 30 days [12, 13].

## Conclusions

Our research study is unique in that it focuses on a specific mental health disorder: severe depression and identifies certain risk factors for 30-day readmission rates in PLWHIV. It is important to assess for risk factors in this population group since improved prognosis in PLWHIV could lessen the severity of medical and mental health burdens.

Risk factors such as age, ethnicity, and access to mental health care in underserved populations, medication adherence, and geographic location can play a pivotal role in improving outcomes like readmission rate within 30 days of discharge, length of stay, etc. Furthermore, vigilance for these risk factors and screening for severe depression in PLWHIV may help to reduce financial burden, hospitalizations, mortality, and morbidity. However, further research is needed - particularly, a prospective model may be better at establishing the cause-effect relationship. 
